# Damage accrual and mortality over long-term follow-up in 300 patients with systemic lupus erythematosus in a multi-ethnic British cohort

**DOI:** 10.1093/rheumatology/kez292

**Published:** 2019-08-03

**Authors:** Beatriz Tejera Segura, Brett Sydney Bernstein, Thomas McDonnell, Chris Wincup, Vera M Ripoll, Ian Giles, David Isenberg, Anisur Rahman

**Affiliations:** Centre for Rheumatology Research, Division of Medicine, University College London, London, UK

**Keywords:** Systemic lupus erythematosus, damage, mortality

## Abstract

**Objective:**

Damage in patients with systemic lupus erythematosus is irreversible change in organs due to disease activity, concomitant disease or medication side-effects. It is measured using the Systemic Lupus International Collaborative Clinics Damage Index (SDI) and is associated with increased mortality. Previous reports have suggested associations between damage accrual and various ethnic, disease and treatment factors, but there is a dearth of long-term follow-up data from large multi-ethnic cohorts. We describe a study of damage and mortality in 300 patients from London, UK followed for up to 40 years.

**Methods:**

We carried out retrospective analysis of medical records and SDI scores of 300 patients followed for up to 40 years (median 13.3 years). Characteristics of the groups who did and did not develop damage and those who died or survived to the end of follow-up were compared using univariable and multivariable analysis. Kaplan-Meier analysis was used to analyse factors affecting mortality and accrual of damage.

**Results:**

Damage developed in 231/300 (77%) of patients. There was a linear accrual of damage over 40 years follow-up. Factors associated with damage were African/Caribbean ethnicity, renal and cerebral involvement, early use of high-dose corticosteroids or immunosuppressants, anti-RNP and antiphospholipid antibodies. Damage was strongly associated with mortality. Of 87 patients who died, 93% had damage compared with 70% of survivors (*P* < 0.001).

**Conclusion:**

Development of damage is strongly associated with increased mortality. We identified groups at increased risk of developing damage, including those treated with high-dose steroids and immunosuppressants within the first two years.

**Rheumatology key messages**

- Long-term follow-up showed linear accrual of damage in 77% of patients with SLE.
- Development of damage in SLE patients was strongly associated with increased risk of death.
- Renal and cerebral involvement, early high-dose corticosteroids and immunosuppressants were associated with increased damage.

## Introduction

SLE is a chronic multi-system autoimmune disease that can affect various organs or systems leading to a broad spectrum of clinical manifestations. These manifestations range from mild and transient to severe and life-threatening. With the introduction of new forms of therapy including immunosuppressive agents and biologics, there has been a considerable improvement in the 5-year survival rate of patients with SLE from 50% to over 90% [1]. Therefore, patients are living longer with the disease and this longer life expectancy can be associated with development of long-term chronic organ damage and disability as a result of persistent disease activity and/or treatment side effects [2]. There is a significant association between the development of damage and mortality in patients with SLE.

To assess chronic organ damage in SLE, the SLICC/ACR damage index (SDI) has been used since 1996 [3, 4]. This index evaluates 12 organs/systems detecting damage in patients regardless of its origin (caused by disease activity or by drug side effects). Each item has to be present for at least 6 months, to differentiate and avoid confusion between disease activity and damage.

Previous cohort data found a higher mortality in patients with SLE developing chronic damage within one year from disease onset [5,6]; however, many of the previous longitudinal studies quantified accrual of damage only at one point during the disease course [7,8].

In a previous paper [9], we reported on SLICC-DI scores in 350 patients from the lupus clinic at University College Hospital London followed for a median of 9 years. The specific aim of that analysis was to assess the impact of factors such as disease activity, therapy and serology recorded during a specific 12-month window after the first clinic visit upon future development of damage. However, that limited analysis did not assess serology and therapy over the whole course of the disease and only 40% of the patients had developed any damage by the end of the follow-up period.

Therefore, in the current paper, we carried out a more comprehensive analysis of factors affecting development of damage in 300 patients with SLE seen at University College Hospital London, with follow-up extending up to 40 years and including data on serological and treatment factors recorded over the whole disease course.

## Methods

### Patients

The study cohort consisted of 300 patients attending the Lupus Clinic of University College Hospital London, who all fulfilled the 1997 revised criteria of the ACR for the diagnosis of SLE [9]. Medical records of all the patients were reviewed to identify demographic data, clinical manifestations of SLE and previous and current treatment. Damage was assessed using the SDI. Only data obtained as part of routine clinical management were included and only pooled data with no patient identifiable information are reported in this paper. Thus, research ethics approval was not required.

Among the 300 SLE patients, we identified 231 (77%) patients who had damage and compared their medical records with those of the 69 patients who had never developed damage. By retrospective analysis of medical records, we obtained comprehensive information about demographic, clinical, serological and treatment factors in these two groups.

With reference to the treatment, use of early high-dose steroids was defined as daily dose ⩾5 mg prednisolone started within the first two years after diagnosis. This cut-off was chosen on the basis of data from the Hopkins Lupus Cohort, Baltimore, USA showing significant increase in damage at mean dose >6mg/day [10]. Any i.v. methylprednisolone pulses were also counted as high dose.

### Statistical analysis

Demographic and clinical characteristics were compared between groups of patients – damage *vs* no damage, early damage (⩽5 years) *vs* late damage, and those who died *vs* those who survived: who developed damage and those who did not, using a Pearson χ^2^ for categorical variables or a Student’s t test for continuous variables [data expressed as mean (s.d.)]. For non-continuous variables, either a Mann–Whitney U test was performed or a logarithmic transformation was made, and data expressed as median and interquartile range (IQR). Multivariable logistic regression analysis was performed to establish the relation of demographic, clinical and treatment factors. Survival and damage-free survival from enrolment in the clinic were assessed by the Kaplan Meier method.

The STATA for Windows statistical software package (v.13.1) was used for all statistical analysis. Significance was defined as *P* <0.05.

## Results

Patients who develop damage are more likely to have experienced renal or CNS involvement or to have taken steroids/immunosuppressants than those with no damage.

Table 1 shows a comparison of the 231 patients (77% of the total cohort of 300 patients) who developed chronic damage and the group of 69 patients who have not developed damage. These two groups did not differ in age, sex or ethnicity. 

**Table 1:** Comparison of characteristics of patients with and without damage

	Damage (*n* = 231, 77%)	Non-damage (*n* = 69, 23%)	*P*-value
Age onset SLE, mean (s.d.)	31 (0.71)	30 (1.3)	0.389
Age F-U SLE, mean (s.d.)	40 (10.9)	39 (12.7)	0.494
Time to damage – in months, mean (s.d.)	114 (83.2)	NA	
Mean months F-U with no damage	NA	328 (164.2)	
Female, *n* (%)	217 (93.9)	63 (91.3)	0.441
Ethnicity, *n* (%)			
Caucasian	148 (64.1)	45 (65.2)	0.484
Afro-Caribbean	59 (25.5)	20 (29)	
Asian	24 (10.4)	4 (5.8)	
Skin disease, *n* (%)			
Rash	167 (72.3)	57 (82.6)	0.084
Photosensitivity	99 (42.9)	34 (49.3)	0.346
Alopecia	44 (19)	10 (14.5)	0.387
Mouth ulcers	59 (25.5)	15 (21.7)	0.520
Joint disease, *n* (%)	220 (95.2)	65 (94.2)	0.729
Kidney disease, *n* (%)	97 (42)	11 (15.9)	**<0.001**
Serositis, *n* (%)	115 (49.8)	28 (40.6)	0.179
CNS disease, *n* (%)	70 (30.3)	10 (14.5)	**0.009**
Positive dsDNA, *n* (%)	148 (64.1)	33 (47.8)	**0.016**
Low complement (ever)	106 (45.88)	20 (23)	**0.013**
ENAs, *n* (%)			
SM	27 (11.7)	7 (10.1)	0.723
Ro	79 (34.2)	21 (30.4)	0.561
La	30 (13)	8 (11.6)	0.760
RNP	63 (27.3)	9 (13)	**0.015**
RF	62 (26.9)	15 (21.7)	0.395
APS antibodies, *n* (%)			
Positive	60 (26)	6 (9)	**0.044**
Treatment (ever), *n* (%)			
Steroids (oral, i.v.)	176 (82.5)	34 (58.6)	**<0.001**
HD steroids	121 (70.3)	27 (52.9)	**0.001**
Cyclophosphamide	35 (16.8)	5 (7.6)	0.064
Azathioprine	105 (50.5)	10 (15.2)	**<0.001**
Mycophenolate	48 (23.1)	4 (6.1)	**0.002**
Rituximab	18 (8.9)	1 (1.5)	**0.046**
Hydroxycloroquine	141 (67.8)	36 (54.5)	**0.050**
Early use of HD steroids	103 (59.9)	25 (43.1)	**0.026**
Early use of IS	85 (49.7)	12 (20.7)	**<0.001**

Numbers in bold are the ones with *P*-value < 0.05. F-U: follow-up; HD steroids: high dose of steroids; IS: immunosuppressants; Lupus AC: lupus anticoagulant; NA: not applicable.

Patients who developed damage were more likely to have suffered from kidney involvement than those who did not (42% *vs* 16%, *P* <0.001) and the same was true of central nervous system involvement (30% *vs* 15%, *P* =0.009). No other clinical features differed between the damage and no damage groups. In terms of serology, a history of ever having low complement or elevated anti-dsDNA were both more common in the damage than the non-damage group (46% *vs* 23%, *P* = 0.013; and 64% *vs* 48%, *P* =0.013, respectively). Among the antibodies to extractable nuclear antigens, only the presence of positive anti-RNP antibody was more frequent in the damage than the no damage group (27.3% *vs* 13%, *P* =0.015). Positivity for antiphospholipid antibodies (anti-cardiolipin and/or anti-beta-2-glycoprotein I and/or lupus anticoagulant at any time) was also more frequent in the damage group (29% *vs* 11% *P* =0.044). There was no difference between groups who were single-positive, double-positive or triple-positive for these three antiphospholipid tests.

Patients in the damage group were more likely to have ever been treated with steroids and various immunosuppressants (IS). Whereas 82.5% of the damage group had ever been treated with steroids, this was only true in 58.6% of the non-damage group (Pearson χ^2^ = 13.83, *P*< 0.001). Patients in the damage group were significantly more likely to have been treated with azathioprine (Pearson χ^2^ = 25.68, *P*<0.001), mycophenolate (Pearson χ^2^ = 9.70, *P* = 0.002) or rituximab (Pearson χ^2^ = 3.98, *P* = 0.046), but not cyclophosphamide. Hydroxychloroquine had been taken by 54.5% of the non-damage group compared with 67.8% of the damage group with no significant difference between both groups.

After multivariable analysis, including significant variables in the previous analysis plus age at the diagnosis of SLE and ethnicity, only age at diagnosis of SLE (OR 1.04, *P* =0.014; 95% CI 1.00, 1.08), taking azathioprine ever (OR 5.26, *P* =0.006; 95% CI 1.62, 17.1) kidney disease (OR 4.22; *P* =0.006; 95% CI 1.51, 11.76), CNS manifestations (OR 3.56, *P* =0.007; 95% CI 1.42, 8.95) and positive anti-RNP antibody (OR 3.45 *P* =0.029; 95% CI 1.22, 9.8) remained statistically significant.

### Time course of development of damage

For the whole group of 300 patients, the median follow-up time from diagnosis was 160 months (range 12–468 months). Of the 231 patients who developed damage (SDI ⩾ 1) the mean time to onset of damage was 9.5 years (s.d. 6.97 years) from diagnosis of SLE. SDI was measured at the first, fifth, 10th, 15th, 20th and 25th year after the diagnosis of SLE. The results are shown in Table 2. 

**Table 2:** Distribution of chronic damage in SLE patients

Time(years)	Patients under follow-up	SDI = 0 *n* (%)	SDI = 1 *n* (%)	SDI > 1 *n* (%)	Median damage score (range)	Deceased patients
First	300	261 (87)	35 (11.7)	4 (1.3)	0 (0–2)	0 (0)
Fifth	295	177 (60)	84 (28.5)	34 (11.5)	0 (0–3)	5 (1.7)
10th	271	121 (44.6)	92 (33.9)	58 (21.4)	1 (0–4)	8 (2.7)
15th	224	79 (35.3)	80 (35.7)	65 (29.01)	1 (0–5)	8 (2.8)
20th	157	34 (21.7)	66 (42)	57 (36.3)	1 (0–5)	11 (3.9)
25th	105	15 (14.3)	44 (41.9)	46 (43.8)	1 (0–5)	55 (20.5)

By year 1 of follow-up, 13% of patients already had some damage but only 1.3% had SDI > 1. The proportion of patients with SDI = 1 gradually increased to around one-third at Year 10 and then remained stable. It seems likely that this change occurred because the number of patients developing new damage (i.e. SDI increases from 0 to 1) was balanced by those moving into the SDI > 1 group. The proportion in the SDI > 1 group increased more gradually with time, until it reached about 30% at Year 15. It should also be noted that there are a group of patients who never develop any damage, even at 25 years follow-up. The maximum damage score for any patient in the cohort was 5.

Of the 231 patients who developed damage, 124 (53.7%) did so by 5 years of follow-up and 107 (46.3%) at later times. These groups are compared in Table 3. Univariable analysis showed that the same factors that differed between damage and no-damage groups also differed between the early damage and late damage groups except that photosensitivity was more common in early damage than late damage, whereas antiphospholipid and anti-RNP antibodies did not differ between those groups. In multivariable analysis, however, there were no significant differences between the early damage and late damage groups. 

**Table 3:** Comparison of characteristics of patients with and without early damage

	Early damage (*n* = 124, 53.7%)	Late damage (*n* = 107, 46.3%)	*P*-value
Age onset SLE, mean (s.d.)	30 (10.8)	31 (10.8)	0.250
Age F-U SLE, mean (s.d.)	38 (10.5)	42 (11.1)	**0.028**
Female, *n* (%)	117 (94.4)	100 (93.5)	0.081
Ethnicity, *n* (%)			
Caucasian	71 (57.3)	77 (72)	
Afro-Caribbean	37 (29.8)	22 (20.6)	0.064
Asian	16 (12.9)	8 (7.5)	
Skin disease, *n* (%)			
Rash	96 (77.4)	71 (66.4)	0.061
Photosensitivity	63 (50.8)	36 (33.6)	**0.009**
Alopecia	27 (21.8)	17 (15.9)	0.256
Mouth ulcers	33 (26.6)	26 (19.6)	0.688
Joint disease, *n* (%)	117 (94.3)	103 (96.3)	0.497
Kidney disease, *n* (%)	63 (50.8)	34 (31.8)	**0.003**
Serositis, *n* (%)	59 (47.6)	56(52.3)	0.471
CNS disease, *n* (%)	46 (37.1)	24 (22.4)	**0.016**
Positive dsDNA, *n* (%)	87 (70.2)	61 (57)	**0.038**
Low complement (ever)	65 (52.4)	41 (38.3)	**0.032**
ENAs, *n* (%)			
Sm	14 (11.3)	13 (12.1)	0.839
Ro	45 (36.3)	34 (31.8)	0.471
La	17 (13.7)	13 (12.1)	0.725
RNP	33 (26.2)	30 (28)	0.808
RF	29 (23.4)	33 (30.8)	0.202
APS antibodies, *n* (%)			
Positive	33 (26.6)	27 (25.2)	0.570
Treatment (ever), *n* (%)			
Steroids (oral, i.v.)	86 (69.4)	56 (52.3)	**0.002**
HD steroids	75 (60.5)	46 (43)	**0.006**
Cyclophosphamide	24 (19.4)	11 (10.3)	0.121
Azathioprine	62 (50)	43 (40.2)	0.496
Mycophenolate	31 (25)	17 (15.9)	0.176
Rituximab	11 (8.9)	7 (6.5)	0.681
Hydroxycloroquine	80 (64.5)	61 (57)	0.998
Early use of HD steroids	65 (52.4)	38 (35.5)	**0.011**
Early use of IS	55 (44.4)	30 (28)	**0.017**
Death, *n* (%)	42 (33.9)	39 (36.4)	0.682

Numbers in bold are the ones with *P*-value < 0.05. F-U: follow-up; HD steroids: high dose of steroids; IS: immunosuppressants; Lupus AC: lupus anticoagulant.

**Fig. 1:**
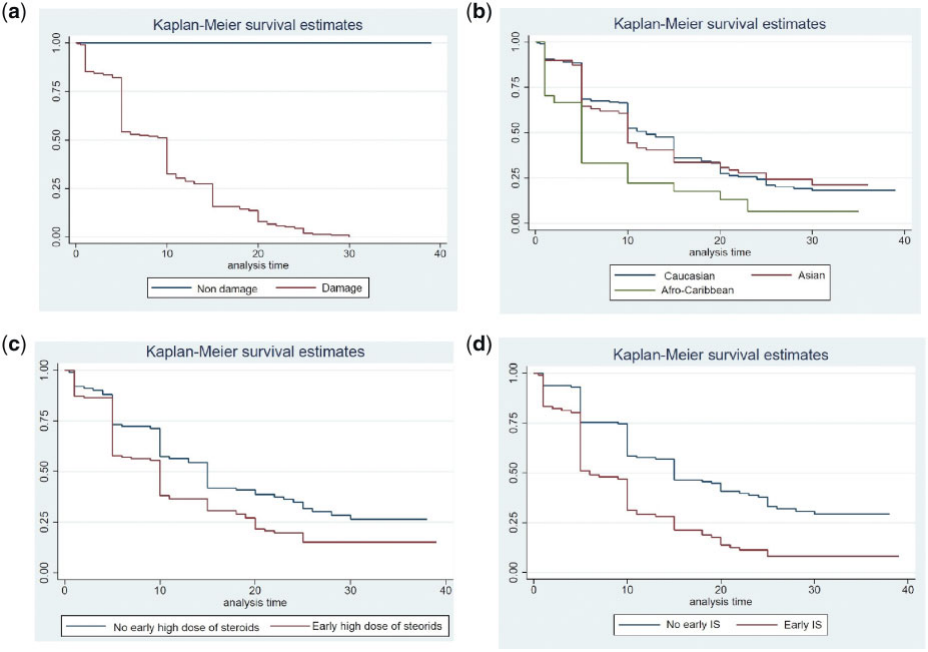
Kaplan-Meier analysis of development of damage in whole population and groups stratified by ethnicity or early therapy (a) Time to damage; (b) ethnicity and damage; (c) early high dose of steroids and damage; (d) early IS and damage.

We used Kaplan-Meier analysis to compare the rates of developing damage in groups stratified according to factors that applied from the beginning of the follow-up period. These were time to damage, ethnicity, early use of high-dose steroids and early use of IS. Kaplan-Meier curves are shown in Fig. 1(a–d). 

Ethnicity was a risk factor for developing chronic damage (HR 1.22 for African/Caribbean patients compared with white patients, *P* =0.045; 95% CI 1.00, 1.50) but there was no risk associated with gender or age at diagnosis of SLE (HR 0.79, *P* =0.402; 95% CI 0.46, 1.36; HR 1.00, *P* =0.932; 95% CI 0.99, 1.01).

With regard to the treatment, taking early high dose of steroids and early use of IS were both risk factors for developing damage (HR 1.47, *P* =0.014; 95% CI 1.08, 1.99; HR 2.08, *P* <0.001; 95% CI 1.53, 2.82).

### Mortality

Overall, 87/300 patients died during the follow-up period. More than a third of the patients (81/231 = 35%) with damage died during their follow-up, whereas only 6/69 (8.7%) patients died in the non-damage group. Conversely 93.1% of patients who died had damage compared with 70.4% of those who survived (*P* <0.001).

Sepsis was the main cause of death (26.4%), followed by cancer (25.3%), cardiovascular events (19.5%), multi-organ failure due to SLE (14.9%) and others (9.2%). There were four patients with unknown cause of death. Table 4 shows the characteristics of the patients who died compared with those who survived to the end of the follow-up period. Patients who died were older at the time of diagnosis of SLE (33.6 years; s.d. ± 12.03 *vs* 29.4 years; s.d. ± 9.77, *P* =0.0006). There were differences among some clinical manifestations between the two groups: CNS disease (Pearson χ^2^ 5.04, *P* =0.025), serositis (Pearson χ^2^ 4.72, *P* =0.030) and alopecia (Pearson χ^2^ 5.91, *P* =0.015) were more common in those who died. 

**Table 4:** Differences between deceased and alive patients

	Deceased (*n* = 87)	Alive (*n* = 213)	*P*-value
Age onset SLE, mean (s.d.)	34 (12.8)	29 (9.7)	**0.0006**
Time to death in months median (IQR)	158 (115)	NA	
SDI>0, *n* (%)	81 (93.1)	150 (70.4)	**<0.001**
Female, *n* (%)	85 (97.7)	195 (91.5)	0.053
Ethnicity			
Caucasian	54 (62.1)	139 (65.3)	0.859
Afro-Caribbean	24 (27.6)	55 (25.8)	
Asian	9 (10.3)	19 (8.9)	
Skin disease, *n* (%)			
Rash	64 (73.5)	160 (75.1)	0.779
Photosensitivity	35 (40.2)	98 (46)	0.361
Alopecia	23 (26.4)	31 (14.6)	**0.015**
Mouth ulcers	25 (28.7)	49 (23)	0.296
Joint disease, *n* (%)	83 (95.4)	202 (94.8)	0.838
Kidney disease, *n* (%)	29 (33.3)	79 (37.1)	0.539
Serositis, *n* (%)	50 (57.5)	93 (43.7)	**0.030**
CNS disease, *n* (%)	31 (35.6)	49 (23)	**0.025**
Positive dsDNA	56 (64.4)	125 (58.7)	0.361
Low complement (ever)	41 (47.1)	85 (39.9)	0.250
ENAs, *n* (%)			
SM	9 (11.1)	25 (11.7)	0.730
Ro	26 (32.1)	74 (34.7)	0.418
La	12 (14.8)	26 (12.2)	0.708
RNP	21 (24.1)	51 (23.9)	0.971
RF	24 (27.6)	53 (24.9)	0.627
APS antibodies			
Positive	28 (32.2)	41 (19.2)	**0.005**
Treatment (ever), *n* (%)			
Steroids (oral, i.v.)	68 (78.2)	108 (50.7)	**0.003**
HD steroids	63 (72.4)	85 (39.9)	**<0.001**
Cyclophosphamide	17 (21.5)	23 (10.8)	0.089
Azathioprine	51(58.6)	64 (30.05)	**<0.001**
Mycophenolate	19 (21.8)	33 (15.5)	0.371
Rituximab	7 (8)	12 (5.6)	0.578
Hydroxycloroquine	49 (56.3)	128 (23)	0.107
Early use of HD steroids	53 (60.9)	75 (35.2)	**0.004**
Early use of IS	36 (41.4)	61 (28.6)	0.338

Numbers in bold are the ones with *P*-value < 0.05. F-U: follow-up; HD steroids: high dose of steroids; IS: immunosuppressants; Lupus AC: lupus anticoagulant; NA: not applicable.

With regard to treatment, patients who died were significantly more likely to have ever taken oral or i.v. steroids (Pearson χ^2^ 9.0, *P* =0.003), to have had high dose of steroids (Pearson χ^2^ 15.34, *P* <0.001), early high dose of steroids (Pearson χ^2^ 8.14, *P* =0.004) and azathioprine (Pearson χ^2^ 16.45, *P* <0.001).

We carried out multivariable analysis, including significant variables in the previous analysis plus age and ethnicity. Age of onset of SLE (OR 1.08, *P* <0.001; 95% CI 1.04, 1.12), having damage (OR 4.08, *P* =0.025; 95% CI 1.19, 14.01), positive antiphospholipid antibodies (OR 3.45, *P* =0.002; 95% CI 1.57, 7.62), serositis (OR 2.22, *P* =0.030; 95% CI 1.04, 4.77), and treatment with azathioprine (OR 2.83, *P* =0.013; 95% CI 1.24, 6.45), remained statistically significant as factors differentiating patients who died from those who survived.

### Survival analysis

**Fig. 2:**
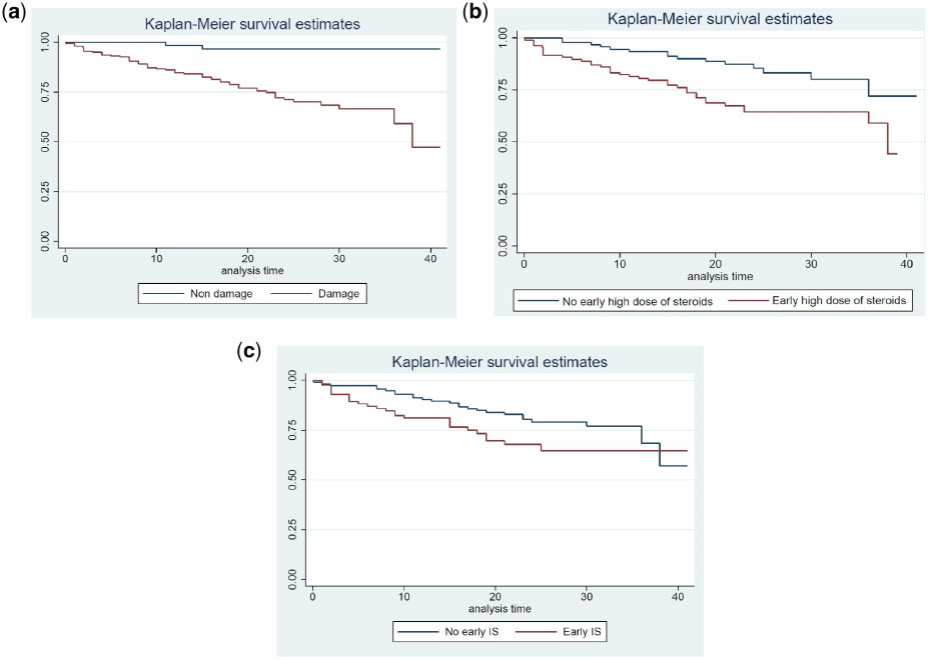
Kaplan-Meier analysis of mortality in whole population and groups stratified by presence of damage or by early therapy (a) Time to death; (b) early high dose of steroids and death; (c) early IS and death.

Figure 2 shows Kaplan-Meier curves analysing factors associated with earlier death in this cohort. The presence of any damage (Fig. 2a) was associated with increased mortality (HR 8.43, *P* <0.001; 95% CI 2.64, 26.9). 

Age at diagnosis, sex, ethnicity, early use of high-dose steroids and early use of IS were analysed. Age at diagnosis (HR 1.04, *P* =0.001; 95% CI 1.02, 1.06) and early use of high dose of steroids (HR 2.48, *P* =0.003; 95% CI 1.35, 4.53) were associated with death. Early IS treatment was not significantly associated with death (HR 1.7, *P* =0.057; 95% CI 0.98, 2.98). After the multivariable analysis, early high dose of steroids remained statistically significant (HR 2.85, *P* =0.001; 95% CI 1.52, 5.34).

## Discussion

In our multi-time-point Kaplan-Meier analysis of a well characterized, multi-ethnic SLE cohort followed for up to 40 years we found development of damage to be strongly associated with increased mortality and identified risk-factors for developing damage in these patients. A number of studies over the last 20 years have investigated factors associated with development of damage in patients with SLE. These include single-centre [2, 6, 10–18] and multicentre [8, 19–25] studies. The reports vary widely in the ethnic composition of the study population, the number of patients and length of follow-up. Some are cross-sectional with SDI measured at a single time point [2, 6, 12, 15, 21, 25] whereas others describe longitudinal SDI data often reported at one-year or five-year time points [16, 18]. Very few reports have used Kaplan-Meier analysis to analyse development of damage (as opposed to mortality) over time [24, 26].

As described by Sutton *et al.* in a systematic literature review of 2013 [4], authors have consistently agreed that increasing age and disease duration are associated with increased SDI. This finding is not surprising given the fact that, by definition, damage is irreversible and the SDI can only increase. For other factors, however, findings are inconsistent between different reports. In some cases, this is because the populations studied do not allow assessment of the effects of particular factors; for example, ethnicity in predominantly monoethnic cohorts [2, 6, 8, 15, 16, 21, 22] or effect of hydroxychloroquine where very few patients are not taking this drug [6, 15]. No previous studies have been able to provide Kaplan-Meier curves for development of damage over 30+ years follow-up, as we have done here. The only studies with similar length of follow-up are from Niigata, Japan [2] and Brescia, Italy [16], both in populations dominated by a single ethnicity. In contrast we report long-term follow-up, including Kaplan-Meier analysis of damage accrual, in a multi-ethnic population.

Those groups who reported on ethnicity generally found that Afro-Caribbean and Hispanic patients develop more damage than Caucasians [4, 14, 19, 24]. Shaharir *et al.* in Malaysia also showed that patients of Indian ethnicity had more damage than Chinese or Malays [12]. However, Petri *et al.* found that the association between Afro-Caribbean ethnicity and damage disappeared on multivariable analysis and concluded that it was confounded by other factors, such as income, hypertension and proteinuria [14]. The curve in Fig. 1b shows that Afro-Caribbean patients in our study do develop more damage than other groups, and the divergence in curves starts early in the disease course. However, this finding is not confirmed by significant *P*-values between ethnic groups in Table 1 or by multivariable analysis, suggesting that ethnicity may be a surrogate for other factors as suggested by Petri [14]. Similarly, Geraldino-Pardilla *et al.* found considerable differences in disease outcomes and damage between two groups of Dominican patients; one in New York City and the other in the Dominican Republic, suggesting that Dominican ethnicity was not the main factor influencing damage [21].

Our study includes only 20 men, which means that we are unable to comment regarding any association of damage with gender (Table 1). Some larger studies have reported that men accrue damage faster than women [24, 27].

Most groups that have measured SDI at various time intervals report a linear increase in the number of patients with damage and the median SDI [4, 15, 16, 18, 24]. An exception is the paper of Nossent *et al.* [25] from a multicentre European cohort, where they suggested that SDI reaches a plateau. It has, however, been argued that this finding could be due to a healthy survivor effect, whereby the patients who would have continued to develop damage died before 20 years of follow-up. In our current study, Fig. 1a supports the idea of linear accumulation of damage with time, with no plateau being seen.

Similar to our findings, other groups have reported an association between damage and CNS involvement [4, 8, 11, 12, 25] or renal disease [2, 4, 15, 17, 25, 28, 29]. Our findings of more damage in patients with elevated anti-dsDNA and reduced complement are also consistent with some reports [2, 12–14, 25] though others found no such link [16, 24]. However, although Prasad *et al.* [13] found that anti-SM positivity was associated with increased development of damage, ours is the first study to report an association between positive anti-RNP antibody and damage. In contrast, the association between antiphospholipid-positivity and damage has been established by many authors [2, 4, 12, 14] and our results support this conclusion.

The association between use of steroids and damage has been widely evaluated in previous reports [4–6, 10, 14–18, 22–25, 27, 30]. The relationship is complex because, whereas these drugs can cause effects such as cataract and avascular necrosis, under-use of steroids in patients with active disease could also lead to damage such as renal failure. Use of steroids has been recorded in different ways, such as ever-use, cumulative dose and average daily dose. The majority of studies have shown that steroids are associated with higher SDI and Al-Sawah *et al.* pointed out that even a reduction of 1 mg per day in average dose could potentially reduce damage [10]. Conti, *et al.* [6] found a strong correlation between glucocorticoids and damage. Gladman and colleagues [31] also attributed the presence of damage to the use of steroids, particularly later in the disease course. Data from the Lupus in Minorities: Nature *vs* Nurture (LUMINA) cohort suggested an association between daily use of glucocorticoids and a shorter time to damage development [23, 27]. A far smaller number of studies did not find a relation between damage and use of steroids, including a very large Japanese study which, similar to ours, included SDI data from 293 patients with up to 40 years follow-up [2].

Data on association of IS use with damage accrual are less extensive, as not all papers report on these drugs and different authors have looked at different drugs. Sutton *et al.* in their literature review described associations between damage accrual and use of cyclophosphamide or azathioprine though pointing out the possibility of confounding by indication (i.e. only those with more severe disease receive these drugs) [4]. Petri *et al.* [14] and Nossent *et al.* [25] found an association with cyclophosphamide but not azathioprine.

In our study, both steroids and some IS (azathioprine, mycophenolate and rituximab but not cyclophosphamide) were associated with damage in the univariate analysis but not in the multivariable analysis. A possible explanation for these findings is due to collinearity of steroid and IS dose so that neither can be shown to exert an independent effect. In an effort to determine the role of therapy decisions taken early in the course of disease, we carried out Kaplan-Meier analysis comparing patients who received high-dose steroids or IS within the first two years of follow-up with those who did not. Figure 1c and d clearly show that patients who receive these treatments early in the disease course develop more damage over time and that the curves remain separate throughout the follow-up period.

In contrast to several previous authors [4, 10, 12, 14, 24] we found no convincing evidence that use of hydroxychloroquine protects against damage development. The major causes of death in our patient group were sepsis, cancer, cardaiovascular events and SLE disease activity which is similar to the results of previous groups [2, 15, 16]. Both Kaplan-Meier analysis (Fig. 2a) and multivariable analysis confirmed the strong association between presence of damage and increased risk of death that has been established in other groups. Early high-dose steroids, but not early IS were associated with increased mortality.

A limitation of our analysis was the absence of data on disease activity. The association of active disease with damage accrual had already been established in this same group of patients by Lopez *et al.* [9] and we did not have activity data covering the whole follow-up period. However, low complement, elevated anti-dsDNA and presence of renal or CNS involvement are all markers of more active disease and all were associated with increased damage. It would also have been interesting to look at individual items of damage to investigate whether particular organs are affected by damage earlier than others. This was beyond the scope of the current analysis.

In conclusion, this study is the first report of damage accrual and mortality in a large multi-ethnic cohort of patients with SLE followed for up to 40 years and underlines the importance of preventing or minimizing damage as far as possible, given the strong link between damage and mortality. Patients who receive high-dose steroids and IS early in the disease course are a group with poorer outcomes as regards damage and mortality. However, it is not clear whether this finding is primarily because of adverse effects of the drugs or because these patients have severe disease from the beginning, requiring increased use of those agents to ameliorate disease. A move to treatment of severe SLE using less toxic drugs may begin to resolve this conundrum as well as achieving better outcomes for patients.
