# Damage accrual and mortality over long-term follow-up in 300 patients with systemic lupus erythematosus in a multi-ethnic British cohort

**DOI:** 10.1093/rheumatology/kez516

**Published:** 2019-10-19

**Authors:** Beatriz Tejera Segura, Brett Sydney Bernstein, Thomas McDonnell, Chris Wincup, Vera M. Ripoll, Ian Giles, David Isenberg, Anisur Rahman

**Affiliations:** Centre for Rheumatology Research, Division of Medicine, University College London, London, UK

Rheumatology 2020;59:524–33. doi:10.1093/rheumatology/kez292

The funding statement, acknowledgements and Disclosure statement were missed from the original article. These have been corrected and appear below. The Publisher apologizes for the errors. Author Vera Ripoll’s middle initial M has also been added.

*Funding:* This work was carried out at a centre supported by the National Institute for Health Research University College London Hospitals Biomedical Research Centre. BTS was supported by the Spanish Foundation of Rheumatology (FER). CW and VR were supported by LUPUS UK. CW is supported by Versus Arthritis Clinical Research Fellowship 21992. VR is supported by the Louise Gergel Fellowship. TM was supported by MRC grant MR/P017371/1.

*Acknowledgements:* We thank Rekha Lopez for assistance with collecting damage data.

*Disclosure statement:* The authors have declared no conflicts of interest.

